# PEG Linker Length Strongly Affects Tumor Cell Killing by PEGylated Carbonic Anhydrase Inhibitors in Hypoxic Carcinomas Expressing Carbonic Anhydrase IX

**DOI:** 10.3390/ijms22031120

**Published:** 2021-01-23

**Authors:** Utpal K. Mondal, Kate Doroba, Ahmed M. Shabana, Rachel Adelberg, Md. Raqibul Alam, Claudiu T. Supuran, Marc A. Ilies

**Affiliations:** 1 Department of Pharmaceutical Sciences, Moulder Center for Drug Discovery Research, Temple University School of Pharmacy, 3307 N Broad Street, Philadelphia, PA 19140, USA; tuf08263@temple.edu (U.K.M.); tug91732@temple.edu (K.D.); tuf15203@temple.edu (A.M.S.); adelberg82@gmail.com (R.A.); tuf06150@temple.edu (M.R.A.); 2 Department of Pharmaceutics and Industrial Pharmacy, Faculty of Pharmacy, Cairo University, Cairo 11562, Egypt; 3 NEUROFARBA Department, Pharmaceutical Sciences Section, Universita degli Studi di Firenze, Polo Scientifico, via Ugo Schiff no. 6, 50019 Sesto Fiorentino (Florence), Italy; claudiu.supuran@unifi.it

**Keywords:** tumor, hypoxia, carbonic anhydrase, inhibitor, polymer conjugate, PEG, sulfonamide

## Abstract

Hypoxic tumors overexpress membrane-bound isozymes of carbonic anhydrase (CA) CA IX and CA XII, which play key roles in tumor pH homeostasis under hypoxia. Selective inhibition of these CA isozymes has the potential to generate pH imbalances that can lead to tumor cell death. Since these isozymes are dimeric, we designed a series of bifunctional PEGylated CA inhibitors (CAIs) through the attachment of our preoptimized CAI warhead 1,3,4-thiadiazole-2-sulfonamide to polyethylene glycol (PEG) backbones with lengths ranging from 1 KDa to 20 KDa via a succinyl linker. A detailed structure−thermal properties and structure–biological activity relationship study was conducted via differential scanning calorimetry (DSC) and via viability testing in 2D and 3D (tumor spheroids) cancer cell models, either CA IX positive (HT-29 colon cancer, MDA-MB 231 breast cancer, and SKOV-3 ovarian cancer) or CA IX negative (NCI-H23 lung cancer). We identified PEGylated CAIs **DTP1K 28**, **DTP2K 23**, and **DTP3.4K 29**, bearing short and medium PEG backbones, as the most efficient conjugates under both normoxic and hypoxic conditions, and in the tumor spheroid models. PEGylated CAIs did not affect the cell viability of CA IX-negative NCI-H23 tumor spheroids, thus confirming a CA IX-mediated cell killing for these potential anticancer agents.

## 1. Introduction

Genetic and epigenetic mutations can transform normal human cells into malignant ones. As the human body ages and normal repair mechanisms recede, the risk of cancer increases, making it currently the second leading cause of death worldwide. The malignant cells lose their normal physiologic functions and contact inhibition and gain characteristics that facilitate their survival, growth, and metastasis [1,2]. Cancer cells grow aggressively, forming tumors in different parts of the body. Often, their aggressive growth outpaces the growth of supporting vasculature, so they become hypoxic [1,2,3,4]. Many aggressive tumors such as breast, colorectal, ovarian, pancreatic, bladder, and other carcinomas are associated with hypoxia and the upregulation and stabilization of hypoxia-inducible factor 1 (HIF-1) [1,2,3,4,5,6,7]. Subsequently, HIF-1 relocates to the nucleus, where it triggers the expression of many proteins required for malignant tumor survival under hypoxic conditions [5,6,7]. Among them are the isozymes of carbonic anhydrase (CA), namely CA IX and CA XII [8,9,10,11].

**Figure 1:**
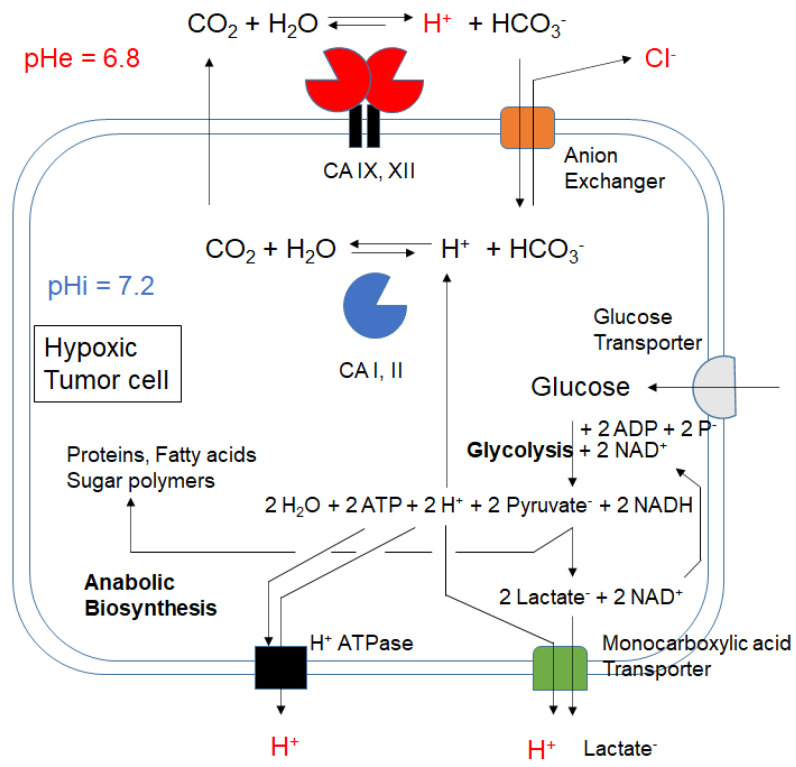
Schematic representation of the central role played by overexpressed membrane-bound carbonic anhydrase (CA) isozymes CA IX and CA XII in tumor metabolism and tumor pH homeostasis: glycolysis is significantly upregulated in tumor cells to make ATP under hypoxic conditions. Protons produced via glycolysis are transported outside the cell via various mechanisms, with cytosolic (CA I and CA II) and membrane-bound CA isozymes (CA IX and CA XII over-expressed in hypoxic tumors) playing a central role. Interfering with tumor pH homeostasis through the inhibition of CA IX and CA XII thus has the potential to control and suppress the proliferation of cancer cells.

Carbonic anhydrases (CAs) are metalloenzymes ubiquitously present in both prokaryotes and eukaryotes, where they serve as catalysts for the reversible hydration of CO_2_ to bicarbonate and a proton. Eight separate gene families encode for these enzymes: α-, β-, γ-, δ-, ζ-, η-, θ-, and ι-CAs [8,12]. The α-CAs are the only group found in mammals, with 14 different CA isozymes being described in humans (hCAs) [8,12]. Of these hCAs, two isozymes are mitochondrial (CA VA and CA VB), five isozymes are present in the cytosol (CA I, CA II, CA III, CA VII, and CA XIII), four isozymes are membrane-bound (CA IV, CA IX, CA XII, and CA XIV), and one isozyme is secreted in saliva and milk (CA VI) [8,12]. Through these isozymes, CA plays an important role in respiration and CO_2_ excretion, pH homeostasis, secretion of electrolytes, gluconeogenesis, lipogenesis and ureagenesis, bone resorption and calcification, and tumorigenicity [8,12]. Focusing on tumorigenicity, it must be emphasized that CA IX and CA XII play central roles in hypoxic tumor cell biochemistry (Figure 1) [6,8,9,13]. A lack of oxygen triggers upregulation of glycolysis as the main source of ATP production, with pyruvate being converted to lactate to regenerate NAD^+^. Glycolysis produces protons which are combined with intracellular HCO_3_^−^ to produce CO_2_ and H_2_O in a reaction catalyzed by cytosolic CAs, especially CA I and CA II. Carbon dioxide passes through the cell membrane, and it is rehydrated outside the cell by overexpressed CA IX and CA XII isozymes to generate HCO_3_^−^ and H^+^. The bicarbonate ion is imported back into the cell and exchanged with Cl^−^ via anion exchangers. The tandem action of cytosolic isozymes with the overexpressed membrane-bound CA IX and CA XII allows the efficient transport of cytosolic protons formed in massive amounts from upregulated glycolysis from inside the tumor cell to the extracellular milieu in an ATP-independent manner. Thus, the pH of the cytoplasm of tumor cells is maintained within normal limits while acidifying the extracellular environment, with detrimental consequences for the normal cells surrounding the tumor (Figure 1) [6,9,13].

Tumor acidification was shown to induce immunosuppression and to contribute significantly to resistance to chemotherapy and radiotherapy [6,9,13,14,15]. CA IX and CA XII expressions were correlated with poor prognosis in many cancers [4,16,17,18,19,20,21,22]. Consequently, inhibition of CA IX and CA XII has the potential to suppress these resistance mechanisms, to re-sensitize the tumors to classical chemotherapeutic drugs, and to induce apoptosis of tumor cells via interference with tumor pH homeostasis [8,9,10,11,15,16,17,20,21,22,23,24,25].

**Scheme 1:**
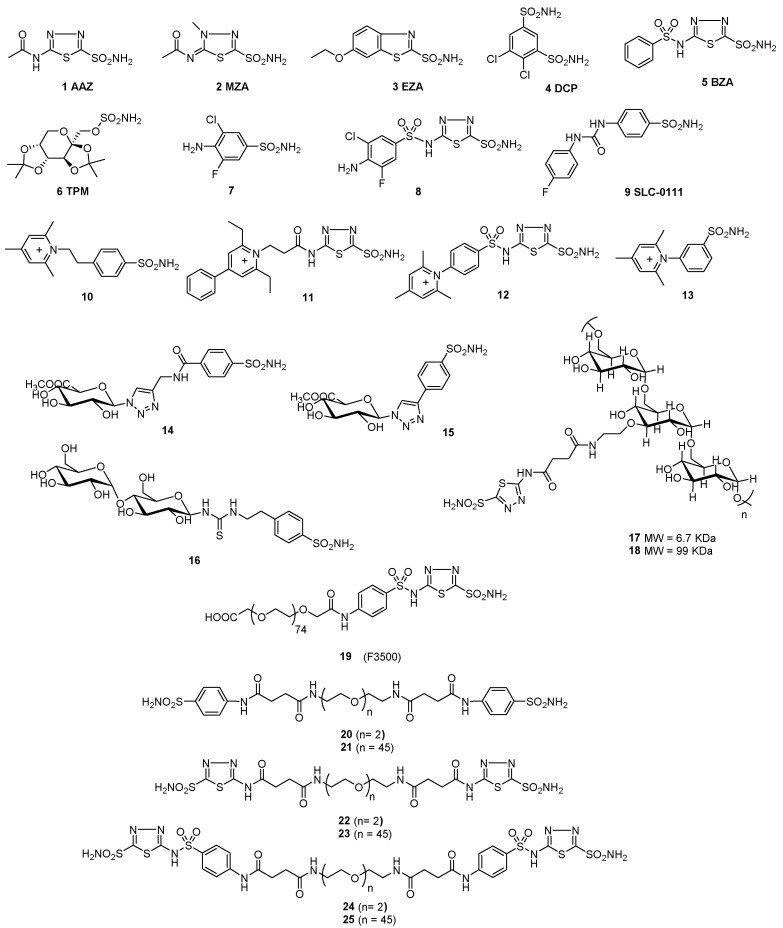
Representative CA inhibitors (CAIs) used in the clinic (**1–6**) and selective CAIs with selectivity against membrane-bound CA isozymes (**7–25**).

Clinically available CA inhibitors (CAIs), such as acetazolamide (**1**, AAZ), methazolamide (**2**, MZA), ethoxzolamide (**3**, EZA), dichlorphenamide (**4**, DCP), benzolamide (**5**, BZA), and topiramate (**6**, TPM, Scheme 1), although potent, lack isozyme selectivity [8]. This translates into high doses of drugs needed to efficiently inhibit tumoral CA IX, which generate significant side effects that prevent their use as anticancer agents. Despite significant medicinal chemistry design efforts that have generated many nanomolar and sub-nanomolar CA IX inhibitors such as **7**, **8**, or **9** (SLC-0111), it is still difficult to generate CAIs that are highly selective for this isozyme due to the high sequence homology and active site similarities between different CA isozymes (Scheme 1) [8,9,17,22,25,26,27,28,29,30].

One of the frequently employed strategies to develop CA IX selective inhibitors is to make the inhibitors membrane-impermeant and thereby to limit their access towards intracellular CA isozymes. Different medicinal chemistry teams, including ours, conjugated known CAI pharmacophores with charged groups (e.g., CAIs **10**–**13**) [31,32,33,34,35,36], with sugar moieties (e.g., CAIs **14**–**16**) [37,38,39,40,41,42,43], or with polymeric backbones (e.g., CAIs **17**–**19**) [44,45] to make the resulting CA inhibitors membrane-impermeant (Scheme 1). For example, representative pyridinium sulfonamides such as **10**, **11**, **12**, and **13**, developed by our team through the conjugation of primary aromatic and heterocyclic sulfonamides with positively charged pyridinium salts, showed excellent selectivity against membrane-bound CA IV [31,32,34,46] and against tumor-associated CA IX and CA XII [35,36,47]. Another alternative design approach for generating membrane-impermeant CA IX selective inhibitors is the conjugation of known CA pharmacophores with water-soluble polymeric backbones such as dextran, aminoethyldextran, and polyethylene glycol (PEG) [44,48]. Three representative inhibitors such as **17–19** (Scheme 1), developed in the late 80s and mid-90s, were impure and polydispersed and showed unwanted side effects when administered in vivo. Interestingly, from the different polymeric backbones tested, polyethylene glycol (PEG) proved to be the most efficient and biocompatible one. Polyethylene glycol conjugation (PEGylation) of drugs also provides several additional benefits such as improved drug solubility; increased stability and circulation time of the drug in the bloodstream; decreased drug immunogenicity, proteolysis, and renal excretion; and optimized drug pharmacokinetics [49].

Building on these premises and taking into account that CA IX and XII are dimers in vivo [50,51], our group developed a series of bifunctional PEGylated CAIs **20–25** by attaching known CAI pharmacophores (aminobenzenesulfonamide, 5-amino-1,3,4- thiadiazole-2-sulfonamide, and aminobenzolamide) on oligoethylene glycol (EG) or on a PEG2000 polymeric backbone (PEG2K) (Scheme 1) [52] All the bifunctional PEGylated and oligoethylene glycol analog CAIs **20–25** were tested for their ability to inhibit off-target cytosolic and membrane-bound isozymes of carbonic anhydrase, including CA IX and CA XII. The results (Table 1) showed an excellent inhibition profile against membrane-bound isozymes CA IX and CA XII, with nanomolar potency of most representatives against these targets. [52]. Interestingly, the long PEG linker increased the selectivity of bis-sulfonamides **21**, **23**, and **25** for the membrane-bound isozymes versus the cytosolic ones (especially against CA II) compared with their oligoethylene glycol congeners **20**, **22**, and **24** (Table 1). Subsequent in vitro testing of oligo/polyEG CAIs **20–25** was performed in 2D under normoxic and hypoxic conditions and in 3D (tumor spheroids) models of cancer. The CAIs were assessed for their ability to reduce the viability of the colon carcinoma HT-29 cell line, the breast cancer MDA-MB 231 cell line, and the ovarian cancer SKOV3 cell line, all expressing CA IX and CA XII isozymes [52,53,54,55,56,57,58,59,60,61,62,63,64,65,66,67,68,69]. The in vitro testing identified PEGylated bis-sulfonamide **23**, bearing the classical 1,3,4-thiadiazole-2-sulfonamide “warhead”, as the most efficient inhibitor, which displayed consistent and significant cancer cell killing at concentrations of 10–100 μM across all three cell lines in 2D/3D cellular models under both normoxic and hypoxic conditions [52]. We assigned this excellent potency against purified CA IX and CA XII isozymes and the consistent and significant cancer cell killing obtained with PEGylated CAI **23** to the cooperative binding and bivalent association of this type of two-pronged inhibitor with the two neighboring active sites [70], as shown by Whitesides [71].

Based on this cooperative binding mechanism, it is expected that PEGylated bis-sulfonamide congeners of **23** having different PEG backbone lengths will display the 1,3,4-thiadiazole-2-sulfonamide warhead differently and will inhibit the dimeric CA IX and CA XII in a different manner. In order to test our working hypothesis, we designed five different PEGylated bis-sulfonamides bearing PEG 1K, PEG 2K, PEG 3.4K, PEG 5K, and PEG 20K polymeric backbones, which were synthesized via our standard procedure and were tested in vitro for their ability to kill cancer cells expressing CA IX and CA XII.

## 2. Results and Discussion

### 2.1. Synthesis of Polymeric CAIs

**Scheme 2:**
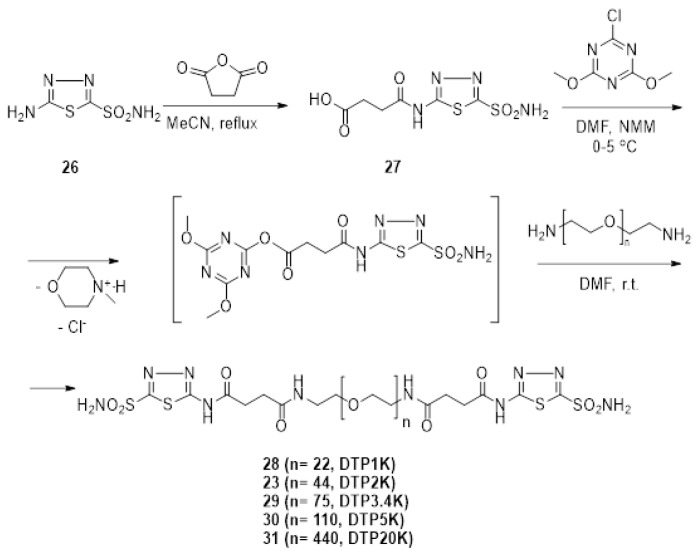
Synthetic strategy for the generation of bis-sulfonamide CA inhibitors.

The new series of compounds **23** and **28–31** were synthesized following previously reported procedure (Scheme 2) [52]. The starting 5-amino-1,3,4-thiadiazole-2-sulfon- amide **26** was synthesized as previously described [31,72,73]. The succinyl derivatives **27** was generated from the condensation of 5-amino-1,3,4-thiadiazole-2-sulfonamide **26** with succinic anhydride in acetonitrile at room temperature. The carboxy group of succinylamido sulfonamide was subsequently activated with 2-chloro-4,6-dimethoxy-1,3,5-triazine (CDMT)/N-methylmorpholine (NMM) [52,74,75] and was condensed with diamino polyethylene glycols having different PEG lengths to yield the bis-sulfonamides **28–31**. These compounds were purified by flash chromatography and characterized by standard analytical methods (see the Materials and Methods section).

### 2.2. DSC Analysis of Polymeric CAIs

**Figure 2:**
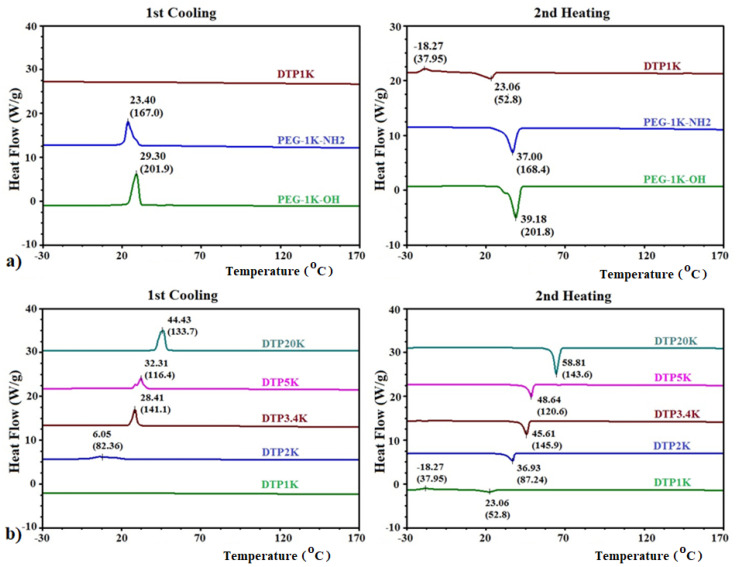
Comparative differential scanning calorimetry (DSC) thermograms (5 °C/min heating/cooling rate) of (**a**) polyethylene glycol (PEG)-1K-OH and PEG-1K-NH2 versus PEGylated bis-sulfonamide DTP1K **28** and of (**b**) PEGylated bis-sulfonamides DTP2K **23** and DTP1K **28**–DTP20K **31**: values in parentheses are the integrated heat signals. Tc and Tm were heating/cooling-rate dependent (data not shown).

Differential scanning calorimetry (DSC) is a powerful method to assess the purity of polymers and polymer conjugates and to characterize the thermal behavior of these chemical entities in bulk, which are important for pharmaceutical formulations containing them [76]. Consequently, DSC was used to analyze the thermal properties of the new PEGylated bis-sulfonamides DTP2K **23** and DTP1K **28**–DTP20K **31** (Figure 2). DSC thermograms of polyethylene glycol 1000 (PEG-1K-OH) and polyethylene glycol 1000 diamine (PEG-1K-NH2) were also presented for comparison.

DSC thermograms from Figure 2a in the first cooling cycle and second heating cycle (5 °C/min heating/cooling rate, respectively) revealed that polyethylene glycol 1000 (**PEG-1K-OH**) has a sharp phase transition with a crystallization temperature Tc of 29.30 °C and a melting temperature Tm of 39.18 °C. The approximately 10 °C supercooling reflects a relatively easy adoption of a crystalline structure due to the high mobility –OH small end group. A change of the end functional group from –OH to –NH2 in diamino PEG (**PEG-1K-NH2**) slightly affected the stability of the PEG backbone, which presented a Tc at lower temperature (23.40 °C). The Tm (37 °C) was also lower than the one observed for **PEG-1K-OH**, revealing that amino groups slightly destabilize the PEG backbone. The lower stability of **PEG-1K-NH2** compared with **PEG-1K-OH** was also reflected by the lower enthalpy observed during crystallization/melting (≈168 J/g for **PEG-1K-NH2** vs. ≈202 J/g for PEG-1K-OH). The conjugation of warhead 2-sulfonamide-1,3,4-thiadiazole-5-(succinamido)- on PEG significantly destabilized the PEG backbone. PEGylated bis-sulfonamide **DTP1K 28** did not show any phase transition in the first cooling cycle. Upon heating, the glassy material crystallizes at approximately −18 °C and then melted at around 23 °C. The enthalpy observed upon melting was about 1/3 of the corresponding thermal effect observed for **PEG-1K-NH2**, reflecting the strong destabilizing effect of the bulky warhead on the (relatively short) PEG backbone. Elongation of the PEG backbone (Figure 2b) allowed better organization of the PEG linker upon cooling. **DTP2K 23** managed to crystallize upon cooling (with about 30 °C supercooling). Longer PEG linkers (3.4K to 20K) raised the Tc and reduced the supercooling effect monotonously, from ≈17 °C (**DTP3.4K 29**) to ≈16.3 °C (**DTP5K 30**) to ≈14.4 °C (**DTP20K 31**). It appears that once the linker length reached a critical length (≈3.4K), the impact of the terminal warheads on the backbone is minimized and the two terminal warheads can be considered isolated from one another. Tm increased monotonously from 36.93 °C (**DTP3.4K 29**) to 58.81 °C (**DTP20K 31**), as expected. Interestingly, the crystallization/melting enthalpy did not follow a linear trend. **DTP3.4K 29** displayed the highest thermal effect (≈142 J/g), followed by **DTP20K 31** (≈140 J/g) and **DTP5K 30** having an intermediate value (≈118 J/g). This trend probably reflects the different relative positions of the two warheads and the impact of PEG length and coiling towards their separation. We postulate that the two warheads are relatively well separated in **DTP3.4K** and **DTP20K** and can come closer to each other in **DTP5K**, thus hampering crystallization and reducing the enthalpy associated with it.

### 2.3. Biological Testing of Polymeric CAIs

**Figure 3:**
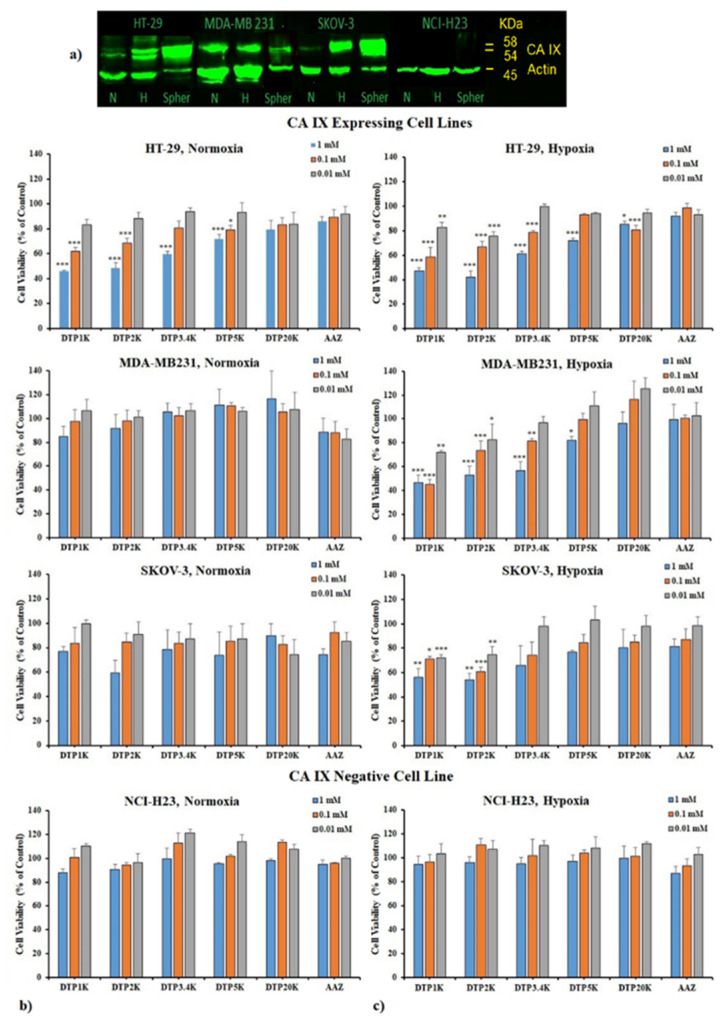
(**a**) Expression of CA IX target in 2D (normoxic/hypoxic) and 3D cancer cell cultures and the effect of PEGylated CAIs **DTP2K 23** and **DTP1K 28–DTP20K 31** and acetazolamide **AAZ 1** at different concentrations on the viability of CA IX expressing cell lines (HT-29, MDA-MB231, and SKOV-3) and CA IX-negative cell line (NCI-H23) under normoxic (**b**) and hypoxic (**c**) conditions (all 2D cell cultures): the *p* values were determined by one-way ANOVA, comparing the value with acetazolamide **1** (* *p* < 0.05, ** *p* < 0.01, and *** *p* < 0.001). Only the statistically significant differences were indicated.

All of the PEGylated bis-sulfonamides **DTP2K 23** and **DTP1K 28**–**DTP20K 31** were tested in 2D and 3D (tumor spheroids) cancer cell models, either expressing CA IX (HT-29 colon cancer, MDA-MB 231 breast cancer, and SKOV-3 ovarian cancer) or CA IX-negative cells (NCI-H23 lung cancer) [52,53,54,55,56,57,58,59,60,61,62,63,64,65,66,67,68,69]. The expression of CA IX under normoxic and hypoxic conditions was previously validated through western blotting (Figure 3a) [52,53,54,55,56,57,58,59,60,61,62,63,64,65,66,67,68,69].

In the 2D cell viability assay, cells were grown sub-confluent in Petri dishes and then plated in 96-well plates in normal conditions (37 °C, 5% CO_2_ in air). For each cell line, half of the plates were incubated in normal (normoxic) conditions, while the other half were subjected to a hypoxic condition (1% O_2_, 5% CO_2_, and 94% N_2_) to induce the expression of the CA IX isozyme. Subsequently, the plates were treated with CAI solutions in media for 24 h of incubation time at three different concentrations (1 mM, 100 µM, and 10 µM) in either normoxic/hypoxic conditions. Cell viability was measured using an 3-(4,5-dimethylthiazol-2-yl)-2,5-diphenyltetrazolium bromide (MTT) assay (Figure 3b,c) [77].

The data from Figure 3 show that the ability of the new PEGylated CAIs to kill the tumor cells correlated well with CA IX expression. All PEGylated CAIs affected tumor cell viability in CA IX expressing cancer cell lines, with a greater effect observed under hypoxic conditions, where CA IX is overexpressed.

Under normoxic conditions, the viability of the HT-29 cell line was significantly reduced after treatment with bis-sulfonamides **DTP2K 23** and **DTP1K 28**–**DTP20K 31**, with CAI having shorter PEG backbones being more efficient. Bis-sulfonamide **DTP1K 28** reduced the cell viability to around 45% and was the best inhibitor in these conditions for this cell line. The order of cell killing efficiency was **DTP1K 28** > **DTP2K 23** > **DTP3.4K 29** > **DTP5K 30** > **DTP20K 31** > **AAZ 1**. The breast cancer MDA-MB231 and ovarian cancer SKOV-3 cell lines were less sensitive to bis-sulfonamide treatment under normoxic conditions, with **DTP1K 28** and **DTP2K 23** being again the most efficient, with an effect matching that of **AAZ 1**. Likewise, the viability of CA IX-negative cell line NCI-H23 was not affected after treatment with these CAIs, implying a CA IX-mediated cell killing for these inhibitors.

However, under hypoxic condition, all three CA IX expressing cell lines were significantly affected by the CAI treatment in a concentration-dependent manner, as expected, with the colon HT-29 and breast MDA-MB231 cancer cells being slightly more sensitive to CAI treatment than ovarian cancer cell line SKOV-3. All the PEGylated CAIs **DTP2K 23** and **DTP1K 28–DTP20K 31** showed significant tumor cell killing abilities. The cell killing efficiency decreased as the length of the PEG linker increased. PEGylated CAIs **DTP1K 28**, **DTP2K 23**, and **DTP3.4K 29,** bearing short and medium PEG backbones, were the most efficient in all three CA IX expressing cell models. The PEGylated bis-sulfonamide **DTP1K 28** was found to be the best inhibitor against all three cell lines under hypoxic conditions, followed by **DTP2K 23** and **DTP3.4K 29.** Both **DTP1K 28** and **DTP2K 23** displayed robust cell killing, with cell viabilities as low as 30–40% at 1 mM, 50% at 100 µM, and 70–80% at 10 µM, being influenced by the cell type and presence of CA IX in large amounts due to hypoxia. On the other hand, inhibitors **DTP5K 30** and **DTP20K 31**, having long PEG linkers, were generally less efficient, even at high concentrations. Inhibitor **DTP5K 30** showed significant cell killing only at 1 mM on the HT-29 cell line and the MDA-MB231 cell line under hypoxic conditions, with inhibitor **DTP20K 31** significantly reducing the viability of the HT-29 cell line only at 1 mM.

All the PEGylated CAIs **DTP2K 23** and **DTP1K 28**–**DTP20K 31** showed superior cell killing profiles to the positive control **AAZ 1** used in this study, which was found to be less efficient toward tumor cell killings under both hypoxic and normoxic conditions. **AAZ 1** was efficient only at very high concentrations, thus revealing the high impact of the PEG backbone on the efficiency of cell killing. Importantly, the CA IX-negative cell line NCI-H23 did not respond to the treatment with these PEGylated CAIs, even under hypoxic conditions.

**Figure 4:**
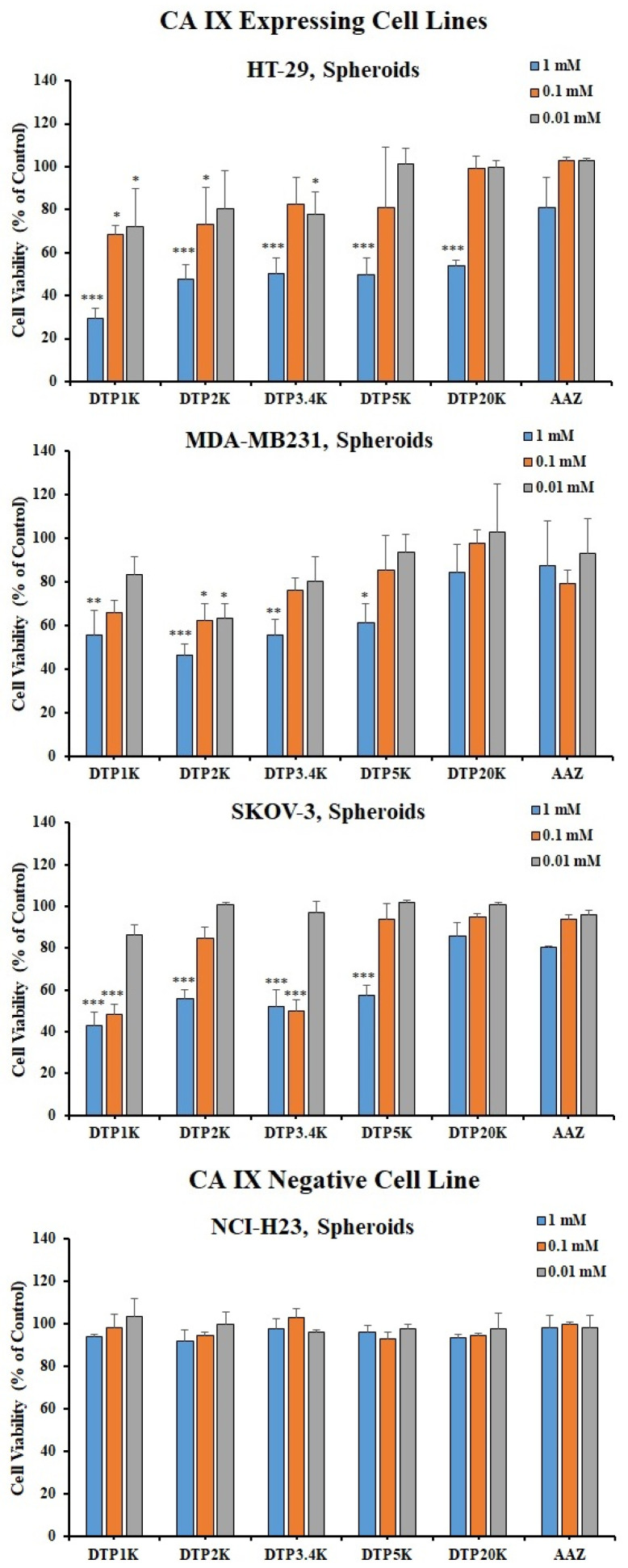
Effect of PEGylated CAIs **DTP2K 23** and **DTP1K 28**–**DTP20K 31** and acetazolamide **AAZ 1** at three different concentrations on the viability of CA IX expressing HT-29, MDA-MB231, and SKOV-3 and of CA IX-negative NCI-H23 3D cell cultures (all 3D tumor spheroids): the *p* values were determined by one-way ANOVA, comparing the value with that of acetazolamide **1** (* *p* < 0.05, ** *p* < 0.01, and *** *p* < 0.001). Only the statistically significant differences are shown.

After evaluating the relative amount of CA IX in tumor spheroids (Figure 3a), we assessed their susceptibility to treatment with CAIs **DTP2K 23** and **DTP1K 28**–**DTP20K 31** at 1 mM, 100 µM, and 10 µM for 24 h of incubation time. Viability of the spheroids was assessed via a water-soluble tetrazolium salt-1 (WST-1) assay (Figure 4) [78].

The cell viability data shown in Figure 4 correlated well with the level of CA IX expression in tumor spheroids (Figure 3a), similarly to the 2D cell cultures (Figure 3b,c). Tumor spheroids become hypoxic due to their 3D growth that limits oxygen perfusion into the spheroid cores. Therefore, the tumor spheroid model is closer to the in vivo conditions. The higher level of expression of CA IX in HT-29 and SKOV-3 tumor spheroids allows one to establish the structure-activity relationship (SAR) for the new PEGylated CAIs even at low inhibitor concentrations of 100 µM and 10 µM. Among the three CA IX expressing cell line, HT-29 and SKOV-3 tumor spheroids were found to be most sensitive to bis-sulfonamide CAI treatments, in good correlation with the CA IX expression level, with the MDA-MB231 cell line being slightly less affected. All the PEGylated CAIs impacted the viability of tumor spheroids for all three CA IX expressing 3D cell cultures, with cell killing efficiency decreasing with an increase in PEG linker length. At high concentrations, all CAIs were efficient in reducing cell viability. At lower concentrations, the shorter CAIs were again more efficient. Thus, PEGylated bis-sulfonamide CAIs having shorter and medium PEG linker **DTP1K 28, DTP2K 23**, and **DTP3.4K 29** were the most efficient CAIs in this assay. PEGylated CAI **DTP1K 28** at 1 mM/100 µM/10 µM significantly decreased the cell viability to about 30%/68%/72% in HT-29, 40%/50%/86% in SKOV-3, and 55%/70%/83% in MDA-MB231, whereas for **DTP2K 23** treatment at the respective concentrations, cell viability decreased to about 50%/75%/80% in HT-29, 55%/85%/100% in SKOV-3, and 45%/62%/64% in MDA-MB 231. High molecular weight PEGylated CAIs **DTP5K 30** and **DTP20K 31** were efficient in killing the cells only at higher concentrations. At 1 mM, CAI **DTP5K 30** decreased the cell viability to 49% in HT-29, 57% in SKOV-3 and to 61% in MDA-MB231 tumor spheroids, while PEGylated CAI **DTP20K 31** only significantly impacted the viability of HT-29 tumor spheroids, expressing the highest level of CA IX. Thus, the length of the PEG linker plays a significant role in killing tumor spheroid cells, with short PEG 1K and 2K linkers being the most efficient for this type of CAIs. Again, all the new PEGylated CAIs exhibited better cell killing efficiency than the positive control **AAZ 1**, which was found to reduce cell viability to about 80% (for 1 mM) and was ineffective at lower concentrations.

Importantly, PEGylated CAIs **DTP2K 23** and **DTP1K 28–DTP20K 31** did not affect the cell viability of CA IX-negative NCI-H23 tumor spheroids, thus reconfirming CA IX-mediated cell killing by these inhibitors.

These data indicate that the length of the PEG backbone plays an important role in tumor cell killing, with the PEG linker significantly affecting the availability of sulfonamide warhead groups for binding into the active sites of dimeric membrane bound CA IX isozyme. Considering a proposed cooperative binding of the bifunctional inhibitor to the two active sites and the fact that PEG may adopt a coiled structure, it is conceivable that a longer linker might place one warhead towards the interior of the coiled backbone, thus decreasing its availability in binding to the dimeric CA IX and therefore lowering the potency, both in vitro and in vivo. Based on the crystal structure of the dimeric CA IX [51], one can calculate that even the PEG 1K is long enough to allow positioning of the two warheads of the PEGylated bis-sulfonamide in the two individual active sites of the CA IX dimer. This cooperative mechanism of action may be different from the interaction of CA IX with classical monodentate inhibitors [8,26,79,80] and warrants further investigations.

## 3. Materials and Methods

Materials. The following materials were used as received: succinic anhydride (TCI America, Portland, OR), N-methylmorpholine (TCI America, Portland, OR, USA), 2-chloro-4,6-dimethoxy-1,3,5-triazine (Acros Organics, New Jersey, NJ, USA), H2N-PEG1000-NH2, H2N-PEG2000-NH2, H2N-PEG3400-NH2, H2N-PEG5000-NH2, and H2N-PEG20,000-NH2 (Laysan Bio, Arab, AL, USA). Other solvents (HPLC quality) were purchased from Fisher Scientific (Pittsburgh, PA, USA), EMD (Gibbstown, NJ, USA), and VWR International (West Chester, PA, USA). Human colon adenocarcinoma cell line (HT-29), human ovary cancer cell line (SKOV-3-Luc), human breast cancer cell line (MDA-MB-231-Luc) and lung cancer cell line NCI-H23 were purchased from ATCC (Manassas, VA, USA). Dulbecco’s Modified Eagle’s medium (DMEM), RPMI-1640, and McCoy’s 5A media were from Mediatech-Corning (Manassas, VA, USA). Fetal bovine serum (FBS) was from Fisher Scientific (Pittsburgh, PA, USA), and 3-(4,5-Dimethylthiazol-2-yl)-2,5-diphenyltetrazolium bromide (MTT), phosphate-buffered saline (PBS), dimethyl sulfoxide (DMSO) and water-soluble tetrazolium salt-1 (WST-1) were purchased from VWR International (West Chester, PA, USA).

Techniques. The purity and structural identity of the intermediates and of the final products were assessed by a combination of techniques that included thin-layer chromatography (TLC), HPLC-MS, ^1^H-NMR, COSY, ^13^C-NMR, high resolution mass spectrometry (HR-MS), and gel permeation chromatography (GPC). TLC was carried out on SiO_2_-precoated aluminum plates (silica gel with F254 indicator; layer thickness 200 µm; pore size 60 Å, from Sigma-Aldrich (St. Louis, MO, USA)).

The purity and the structure identity of the intermediates and of the final products were assessed by a combination of techniques that included thin-layer chromatography (TLC), HPLC-MS, ^1^H-NMR, COSY, ^13^C-NMR, high resolution mass spectrometry (HR-MS), and gel permeation chromatography (GPC). TLC was carried out on SiO_2_-precoated aluminum plates (silica gel with F254 indicator; layer thickness 200 µm; pore size 60 Å, from Sigma-Aldrich).

HPLC-MS was performed using an Agilent 1200 HPLC-DAD-MS system equipped with a G1315A DAD and a 6130 Quadrupole MS using a ZORBAX SB-C18 column, eluted with H_2_O (0.1% HCOOH)/MeCN (0.1% HCOOH) 95/5 to 0/100 linear gradient. Analytical GPC was performed for the polymeric compounds using a Shimadzu Prominence UFLC equipped with a vacuum degasser, CC20AD pump, CBM 20A controller, CTO–20 A column over, and UV and RI detectors, under the control of EZ start software. Separation was performed with Phenomenex Phenogel columns (1 × Phenogel Guard column, 5 µm, linear, 50 × 7.8 mm + 1 × Phenogel 5 µm, 100 Å, 300 × 7.8 mm + 1 × Phenogel 5 µm, 500 Å, 300 × 7.8 mm) eluted with dimethylformamide (DMF) at 50 °C at a flow rate of 1 mL/min. Calibration was done with 10 PEG pure standards, with molecular weights ranging from 200 to 50,000.

NMR spectra were recorded at ≈300 K with a Bruker Avance III 400 Plus spectrometer equipped with a 5-mm indirect detection probe operating at 400 MHz for ^1^H-NMR and at 100 MHz for ^13^C-NMR. Chemical shifts are reported as δ values, using tetramethylsilane (TMS) as an internal standard for proton spectra and the solvent resonance for carbon spectra. Assignments were made based on chemical shifts, signal intensity, COSY, HMQC, and HMBC sequences. For ^1^H-NMR, data are reported as follows: chemical shift, multiplicity (s = singlet, d = doublet, t = triplet, sep = septet, and m = multiplet), coupling constants J (Hz), and integration.

Differential scanning calorimetry (DSC) was performed using a TA Instruments Q200 MDSC (New Castle, DE, UK) and a heating/cooling rate of 5 °C/min for all compounds.

### 3.1. Synthesis of 4-oxo-4-((5-sulfamoyl-1,3,4-thiadiazol-2-yl)Amino)Butanoic Acid 27

In a flame-dried 20-mL reaction vial, 5-amino-1,3,4-thiadiazole-2-sulfonamide **26** (0.45 g, 2.5 mmol) was dissolved in 10 mL of acetonitrile. Succinic anhydride (275 mg, 0.275 mmol) was added over it. The reaction mixture was stirred for 48 h and monitored by TLC and LC-MS. Once the reaction reached completion, the solvent was evaporated to dryness by rotavap. A volume of 10 mL of water was added over the mix to destroy all excess succinic anhydride, to dissolve resulting acid, and to precipitate the product. The precipitate was filtered and rinsed with cold methanol several times to remove water. The precipitate was 98% pure (LC-MS). Yield: 83%; mp. 232–236 °C; lit. [52] m.p. 232–236 °C. ^1^H-NMR (400 MHz, DMSO-d^6^, δ, ppm): 13.06 (br s, 1H, –NH), 12.27 (br s, 1H, NH), 8.32 (s, 2H, –SO_2_NH_2_), 2.76 (t, *J* = 6.5 Hz, 2H, CH_2_COOH), 2.60 (t, *J* = 6.5 Hz, 2H, CH_2_CONH); ^13^C-NMR (100.6 MHz, DMSO-d^6^, δ, ppm): 173.3 (CONH), 171.4 (COOH), 164.1 (C_5_ TDA), 161.0 (C_2_ TDA), 29.9 (–CH_2_COOH), 28.2 (–CH_2_CONH-); LC-MS: C_6_H_8_N_4_O_5_S_2_, exact mass: 280.0; found: 281.0 (MH^+^).

### 3.2. General Procedure for the Preparation of Bis-Sulfonamides 23 and 28–31

In a flame-dried 20-mL reaction vial, succinylamidosulfonamide **27** (0.536 g, 1.92 mmol) and 2-chloro-4,6-dimethoxy-1,3,5-triazine (CDMT, 0.37 g, 2.11 mmol) were dissolved in 2 mL dry DMF. The reaction vial was cooled in an ice bath at 0 °C. After 5 min, N-methylmorpholine (NMM, 235 µL, 0.213 g, 2.11 mmol) was added and the reaction mixture was stirred at 0 °C for 45 min under nitrogen atmosphere. Subsequently, diamino PEG (H_2_N-PEG-NH_2_, 0.500 g, 0.250 mmol), dissolved in 1.5 mL dry DMF, was added dropwise to the reaction mixture. The reaction was stirred at room temperature under nitrogen atmosphere for two days. The DMF was evaporated by rotavap under high vacuum, and the crude compound was absorbed on SiO_2_. Flash chromatography was performed with CHCl_3_/MeOH gradients on a SiO_2_ column. Useful fractions (determined by TLC, performed with DCM/MeOH (75/25 *v*/*v*), and by NMR) were grouped and evaporated to dryness to yield a relatively pure compound which was further purified by another flash chromatography run. Finally, the pure compounds were dried under high vacuum for 24 h.

#### 3.2.1. Bis-((5-Sulfamoyl-1,3,4-thiadiazol-2-yl)-amidosuccinyl)-polyethyleneglycol1000diamide (DTP1K) 28

Yield: 75%; ^1^H-NMR (400 MHz, D_2_O, δ, ppm): 3.65–3.52 (m, 88 H, 22 –OCH_2_CH_2_O–), 3.57–3.52 (m, 4H, –OCH_2_CH_2_NH–), 3.31 (d, J = 7.1 Hz, 4H, –OCH_2_CH_2_NH–), 2.87–2.79 (m, 4H, –COCH_2_CH_2_CO–), 2.66–2.60 (m, 4H, –OCCH_2_CH_2_CO–). GPC: 96%+, t_R_ = 15.86 min.

#### 3.2.2. Bis-(5-Sulfamoyl-(1,3,4)-thiadiazol-2-yl)-amidosuccinyl)-polyethyleneglycol2000diamide (DTP2K) 23

Yield 56.7%; ^1^H-NMR (D_2_O, δ, ppm): 3.80–3.58 (m, 176 H, 44 OCH_2_CH_2_O), 3.55 (t, *J* = 5.8 Hz, 4H, –OCH_2_CH_2_NH–), 3.33 (t, *J* = 5.4 Hz, 4H, –OCH_2_CH_2_NH–), 2.87 (t, *J* = 6.6 Hz, 4H, –COCH_2_CH_2_CO–), 2.63 (t, J = 6.6 Hz, 4H, –OCCH_2_CH_2_CO–). GPC: 96%+, t_R_ = 15.38 min.

#### 3.2.3. Bis-(5-Sulfamoyl-(1,3,4)-thiadiazol-2-yl)-amidosuccinyl)-polyethyleneglycol3400diamide (DTP3.4K) 29

Yield: 55%; 1H-NMR (D_2_O, δ, ppm): 3.81–3.58 (m, 299 H, 77 OCH2CH2O), 3.54–3.53 (m, 4H, –OCH2CH2NH–), 3.32 (t, J = 6.4 Hz, 4H, –OCH2CH2NH–), 2.85 (t, J = 6.6 Hz, 4H, –COCH2CH2CO–), 2.61 (t, J = 6.6 Hz, 4H, –OCCH2CH2CO–). GPC: 96%+, t_R_ = 14.57 min.

#### 3.2.4. Bis-(5-Sulfamoyl-(1,3,4)-thiadiazol-2-yl)-amidosuccinyl)-polyethyleneglycol5000diamide (DTP2K) 30

Yield: 60%; 1H-NMR (D_2_O, δ, ppm): 3.82–3.58 (m, 440 H, 113 OCH2CH2O), 3.55–3.53 (m, 4H, –OCH2CH2NH–), 3.32 (t, J = 6.4 Hz, 4H, –OCH2CH2NH–), 2.86 (t, J = 6.4 Hz, 4H, –COCH2CH2CO–), 2.62 (t, J = 6.8 Hz, 4H, –OCCH2CH2CO–). GPC: 96%+, t_R_ = 14.03 min.

#### 3.2.5. Bis-(5-Sulfamoyl-(1,3,4)-thiadiazol-2-yl)-amidosuccinyl)-polyethyleneglycol20000diamide (DTP2K) 31

Yield: 55%; 1H-NMR (D_2_O, δ, ppm): 3.80–3.57 (m, 1760 H, 440 OCH2CH2O), 3.45–3.43 (m, 4H, –OCH2CH2NH–), 3.31 (t, J = 4.8 Hz, 4H, –OCH2CH2NH–), 2.85 (t, J = 6.8 Hz, 4H, –COCH2CH2CO-), 2.61 (t, J = 6.6 Hz, 4H, –OCCH2CH2CO–). GPC: 96%+, t_R_ = 11.80 min.

### 3.3. Viability Evaluation in 2D Cell Cultures

Four cell lines, namely HT-29, MDA-MB-231, SKOV3, and NCI-H23, were cultured at 37 °C and 5% CO_2_ using RPMI-1640, Dulbecco’s Modified Eagle’s medium (DMEM), McCoy’s 5A Medium, and RPMI-1640, respectively, supplemented with 10% fetal bovine serum. When the cells were about 75% confluent in 75-cm^2^ flasks, they were trypsinized with trypsin-EDTA, counted, and plated in 96-well plates at a density of 10^4^ cells/well with a final volume of 200 µL in each well. Two plates were made for each cell line. After 24 h of incubation in a 5% humidified incubator at 37 °C, one plate from each cell line was placed in the hypoxia chamber and purged for 10 min with the hypoxia gas mixture (1% O_2_, 5% CO_2_, balance N_2_). After 1 h, the chamber was purged again with hypoxic gas mixture and placed back in the incubator. After a 24-h incubation in the incubator at 37 °C, both the normoxic and hypoxic plates were retrieved and the media were removed from all wells. Cells were washed with 5% sterile dextrose and treated with different solutions of different concentrations of CAIs **DTP2K 23** and **DTP1K 28–DTP20K 31** in media, all pre-filtered through a 0.2-µm sterile nylon filter. Each assay was done at least in triplicate. After 24 h of incubation time with CAIs, the supernatant was discarded; cells were washed with phosphate-buffered saline (PBS); and after the removal of PBS, each well received 120 µL of MTT/media solution (prepared from 5 mg/mL MTT in sterile PBS, added to media in the ratio 1:6). The plates were incubated for 4 h at 37 °C, the MTT/media was aspirated off, and 150 μL of DMSO was added to each well to dissolve the formazan crystals. The plates were stored in the incubator for 5 min at 37 °C, and absorbance was read at 570 nm using the 630-nm wavelength as reference.

### 3.4. Viability Evaluation in 3D Cell Cultures

Four cell lines, namely HT-29, MDA-MB-231, SKOV3, and NCI-H23, were cultured at 37 °C and at 5% CO_2_ using RPMI-1640, Dulbecco’s Modified Eagle’s medium (DMEM), McCoy’s 5A and RPMI-1640 medium respectively, supplemented with 10% fetal bovine serum. When the cells were about 75% confluent in 75 cm^2^ flasks, they were trypsinized with trypsin-EDTA, counted, and plated in 96-well plates with round bottom at a density of 10^3^ cells/well in a final volume of 100 µL. After 7 days of incubation in a 5% CO_2_ humidified incubator at 37 °C, the plates were treated with the same solutions/complexes of CAI presented above. Each assay was done at least in triplicate. After 24 h of incubation, cells are treated with 10% WST-8 in media. The plate was stored in the incubator for 4 h, and the plate was subsequently read for absorbance at 450 nm with a reference at 650 nm.

### 3.5. CA IX Profiling Using Western Blotting

The HT-29, MDA-MB231, SKOV-3, and NCI-H23 cell lines were screened for the expression of carbonic anhydrase IX in 2D and 3D cell culture under normoxic and hypoxic conditions. Cells were cultured in 60-mm petri dishes at a density of 10^6^ cells under normoxic and hypoxic conditions. The hypoxic conditions were induced as described above by placing the cells into a hypoxic chamber purged with hypoxia gas mixture containing 1% oxygen for 24 h. The chamber was placed in a 37 °C incubator. For normoxic conditions, cells were kept in a regular incubator for 24 h. The next day, all cells were harvested, pelleted, and washed with phosphate buffer saline (PBS). Cells were then lysed with radioimmunoprecipitation assay (RIPA) buffer containing protease inhibitors, and lysates were collected and stored at −20 °C. Total protein concentration was determined using the bicinchoninic acid method (BCA). The cells were also cultured in rounded bottom 96-well plates at a density of 1000 cells/well and were placed in the incubator for 1–2 week to grow into tumor spheroids. After the spheroids were fully developed, they were harvested and lysed as mentioned previously. Western Blot analysis was performed as described [52]. Briefly, cell lysate samples were loaded onto 10% SDS-PAGE precast gels (Bio-rad, 456–8034), and separation was done at 150 V for 2 h. Gels were transblotted onto nitrocellulose membranes for 30 min, and membranes were blocked using the blocking buffer followed by incubation with a specific primary mouse monoclonal antibody for CA IX (M75, Bioscience Slovakia) at 4 °C overnight. After washing, the membranes were incubated with anti-mouse IgG IRDye800CW secondary antibody (Rockland) for 1 h at room temperature. Finally, membranes were detected using an Odyssey image system (Li-Cor, Lincoln, NE, USA). Beta actin was used as a control and was detected using a mouse monoclonal beta actin antibody (Genetex) binding followed by detection with the same IgG IRDye800CW secondary antibody.

## 4. Conclusions

A series of bifunctional PEGylated CAIs **28–31** congeners of previously reported representative **23** was generated following our established synthetic strategy [52]. The series allowed us to test whether the PEG linker separating the two individual warheads elicit significant effects on the biological properties of this type of CAI. DSC data allowed us to conclude that, starting from a 3.4 KDa linker length, the two warheads are spaced enough to have minimum influence on each other and on the PEG backbone. This linker length was found relevant for the ability of CAIs to kill cancer cells in 2D and 3D (tumor spheroids) that express CA IX. Thus, PEGylated CAIs with short linkers were more efficient in killing these cancer cells in vitro than their congeners with long PEG linkers, confirming a strong impact of cooperativity towards CA IX inhibition with these dimeric CAIs. Tumor cell killing was found to be amplified under hypoxia when the expressions of CA IX and CA XII are strongly induced. Also, the polymeric CAIs did not affect the cell viability of CA IX-negative NCI-H23 cells, thus confirming CA IX-mediated cell killing of these inhibitors.

## Figures and Tables

**Table 1:** Inhibition data of human CA cytosolic isoforms hCA I and II and membrane-bound CA IX and XII with oligo/polyEG derivatives **20–25** and the standard sulfonamide inhibitor acetazolamide **1** by a stopped flow CO_2_ hydrase assay [52].

K_I_ (nM) *					
Compound	EG Units	hCA I	hCA II	hCA IX	hCA XII
20	3	221 ± 16	37.1 ± 0.2	2.9 ± 0.1	2.6 ± 0.2
21	46	272 ± 25	1764 ± 45	4.8 ± 0.1	3.2 ± 0.2
22	3	180 ± 12	8.7 ± 0.5	9.6 ± 0.1	5.9 ± 0.4
23	46	225 ± 21	66.6 ± 3.1	2.5 ± 0.2	5.4 ± 0.3
24	3	192 ± 9	8.6 ± 0.4	24.1 ± 0.2	2.8 ± 0.1
25	46	181 ± 9	12.9 ± 1.0	1.7 ± 0.1	3.7 ± 0.1
1		250 ± 12	12 ± 0.8	25 ± 1.7	5.7 ± 0.3

* Mean ± from standard error from 3 different assays by a stopped flow technique. Monomeric (recombinant) human enzymes were used in all cases.

## Data Availability

The data presented in this study are available in the article. Additional data are available from the corresponding author upon request.
